# Exploring the Constraints on Simulated Aerosol Sources and Transport Across the North Atlantic With Island‐Based Sun Photometers

**DOI:** 10.1029/2020EA001392

**Published:** 2020-11-16

**Authors:** Sam J. Silva, David A. Ridley, Colette L. Heald

**Affiliations:** ^1^ Department of Civil and Environmental Engineering Massachusetts Institute of Technology Cambridge MA USA; ^2^ Now at: Pacific Northwest National Laboratory Richland WA USA; ^3^ Now at: California Air Resources Board Sacramento CA USA

## Abstract

Atmospheric aerosol over the North Atlantic Ocean impacts regional clouds and climate. In this work, we use a set of sun photometer observations of aerosol optical depth (AOD) located on the Graciosa and Cape Verde islands, along with the GEOS‐Chem chemical transport model to investigate the sources of these aerosol and their transport over the North Atlantic Ocean. At both locations, the largest simulated contributor to aerosol extinction is the local source of sea‐salt aerosol. In addition to this large source, we find that signatures consistent with long‐range transport of anthropogenic, biomass burning, and dust emissions are apparent throughout the year at both locations. Model simulations suggest that this signal of long‐range transport in AOD is more apparent at higher elevation locations; the influence of anthropogenic and biomass burning aerosol extinction is particularly pronounced at the height of Pico Mountain, near the Graciosa Island site. Using a machine learning approach, we further show that simulated observations at these three sites (near Graciosa, Pico Mountain, and Cape Verde) can be used to predict the simulated background aerosol imported into cities on the European mainland, particularly during the local winter months, highlighting the utility of background AOD monitoring for understanding downwind air quality.

## Key Points

- Sun photometer AOD observations at island sites characterize long‐range transport of aerosol species across the North Atlantic Ocean
- Model simulations suggest that higher elevation observations provide a pronounced signature of anthropogenic and biomass burning aerosol
- These island sites contain information useful for predicting imported aerosol to the European continent

## 1 Introduction

Atmospheric aerosol plays a critical role in the global earth system, strongly impacting climate change, ecosystem function, and human health. These suspended liquid droplets and particles directly influence the global radiative balance by absorbing and scattering radiation and indirectly by influencing cloud formation and radiative properties (e.g., IPCC, 2013; Prospero & Lamb, 2003). They affect global biogeochemistry by fertilizing the terrestrial and oceanic ecosystems through deposition to the surface and scattering of radiation aloft (e.g., Darwin, 1846; N. Mahowald, 2011; Mercado et al., 2009). Inhalation of these particles negatively impacts human health, ultimately leading to millions of premature deaths per year worldwide (Burnett et al., 2018). There is thus a strong need to understand the global distribution and variability of aerosol to better constrain these impacts.

The lifetime of aerosol in the atmosphere is on the order of 1 week, which is sufficiently long to allow for intercontinental transport (Balkanski et al., 1993; Prospero & Lamb, 2003). For example, Prospero and Lamb (2003) found that variability in mineral dust aerosol emissions from Africa can be constrained with observations at an island site downwind in Barbados, across the Atlantic Ocean. Similarly, Ridley et al. (2012) used surface measurements in North America to evaluate the simulated aerosol life cycle of dust exported from Africa in the GEOS‐Chem chemical transport model. Model estimates (e.g., Mahowald et al., 2005, 2008) and in situ observations (e.g., Barkley et al., 2019) demonstrate that this intercontinental transport can be an important source of nutrients to ecosystems far removed. For example, Barkley et al. (2019) observed that emissions from African biomass burning were a large source of nutrients as far downwind as regions of the Amazon rainforest and the Southern Ocean. Further north in the Atlantic, Ancellet et al. (2016) found that aerosol emissions from North American fires have a strong influence on the aerosol abundance over continental Europe. This long‐range transport is not unique to the Atlantic Ocean, as demonstrated by observations of Siberian fire influence on North American atmospheric composition (e.g., Bertschi & Jaffe, 2005; Jaffe et al., 2004). The long‐range transport of aerosol can substantially impact concentrations near the surface, in some cases worsening air quality and limiting attempts to reduce particulate matter exposure (e.g., Bertschi & Jaffe, 2005). Despite these important impacts, direct constraints on the sources and influence of long‐range transport are challenging to obtain using in situ measurements, as aerosol measurement sites are predominately located on land near large confounding local sources (e.g., Martin et al., 2019; Smirnov et al., 2009).

Island sites in the remote oceans are generally removed from these large local sources and can provide valuable information to evaluate these transport processes (e.g., Prospero & Lamb, 2003; Smirnov et al., 2009). While previous studies have investigated the transport of aerosol species westward across the Equatorial Atlantic (e.g., Barkley et al., 2019) or the influence of specific events on transported species (Ancellet et al., 2016), we focus here on the North Atlantic with particular interest in understanding the long‐term sources of transported aerosol to continental Europe. Analyses of in situ observations indicate the importance of long‐range transport in the North Atlantic (e.g., Gallo et al., 2020; Zheng et al., 2020); however, there has been limited exploration and evaluation of model representations of aerosols over this region. Constraining this trans‐Atlantic transport is central to characterizing aerosol source contributions to the degradation of air quality over Europe as well as investigating how these aerosol species influence climate and biogeochemistry over the ocean (e.g., Carslaw et al., 2013; Foltz & McPhaden, 2008; Penner et al., 1994).

To address these open research questions, we use long‐term sun photometer observations of aerosol optical depth (AOD) located on the islands of Graciosa and Cape Verde. Both locations are in the Eastern North Atlantic, with Graciosa off the coast of Portugal and Cape Verde off the coast of Senegal. Sun photometer observations offer potentially continuous, automated remote sensing of atmospheric composition without the sample transport and analysis needed for in situ sampling and are thus highly useful for long‐term atmospheric monitoring. To date, research at the Graciosa location has largely been focused on understanding the links between aerosol concentrations, cloud properties, and precipitation (e.g., Logan et al., 2014; Naud et al., 2018), whereas observations from the Cape Verde location have been used for decades in a wide variety of work, largely related to constraining the abundance and impacts of dust transport from Africa (e.g., Ansmann et al., 2009; Chiapello et al., 1999; Ridley et al., 2014).

In this study, we use the GEOS‐Chem model to explore the spatiotemporal variability in AOD over the North Atlantic as it is modulated by atmospheric transport, aerosol composition, and aerosol source region. We demonstrate the valuable constraints and utility provided by AOD observations on the North Atlantic islands of Graciosa and Cape Verde and the potential value of long‐term sun photometer observations from Pico Mountain. Through a set of simulation experiments, we further investigate the value of these observation sites as predictors imported aerosol to European cities downwind.

## 2 Data Description

### 2.1 GEOS‐Chem Chemical Transport Model

To investigate the abundance, variability, and sources of aerosol over the North Atlantic, we use v11–01 of the GEOS‐Chem chemical transport model (www.geos‐chem.org). The HO_x_‐NO_x_‐VOC gas‐phase scheme coupled with the‐sulfate‐nitrate‐ammonium (SNA) aerosol scheme used here was first described in Park (2004), and aerosol thermodynamics are calculated using the ISORROPIA II thermodynamic module (Fountoukis & Nenes, 2007). The dust scheme in GEOS‐Chem is described in Fairlie et al. (2007), with updates to the size distributions from Zhang et al. (2013), and emissions generated using the DEAD scheme (Zender et al., 2003). Carbonaceous aerosol follows Wang et al. (2014) for black carbon (BC), Pye et al. (2010) and Pye and Seinfeld (2010) for organic aerosol (OA), and Gantt et al. (2015) for marine primary OA. Sea salt aerosol is from Jaeglé et al. (2011) and varies with surface wind speed and ocean temperature. AOD is calculated using local relative humidity and optical properties from Martin et al. (2003), with dust specific properties from Ridley et al. (2012). For numerical integration in this work, we use a time step of 10 min for convection and transport and 20 min for emissions and chemistry and a spatial resolution of 2° × 2.5°.

**Figure 1:**
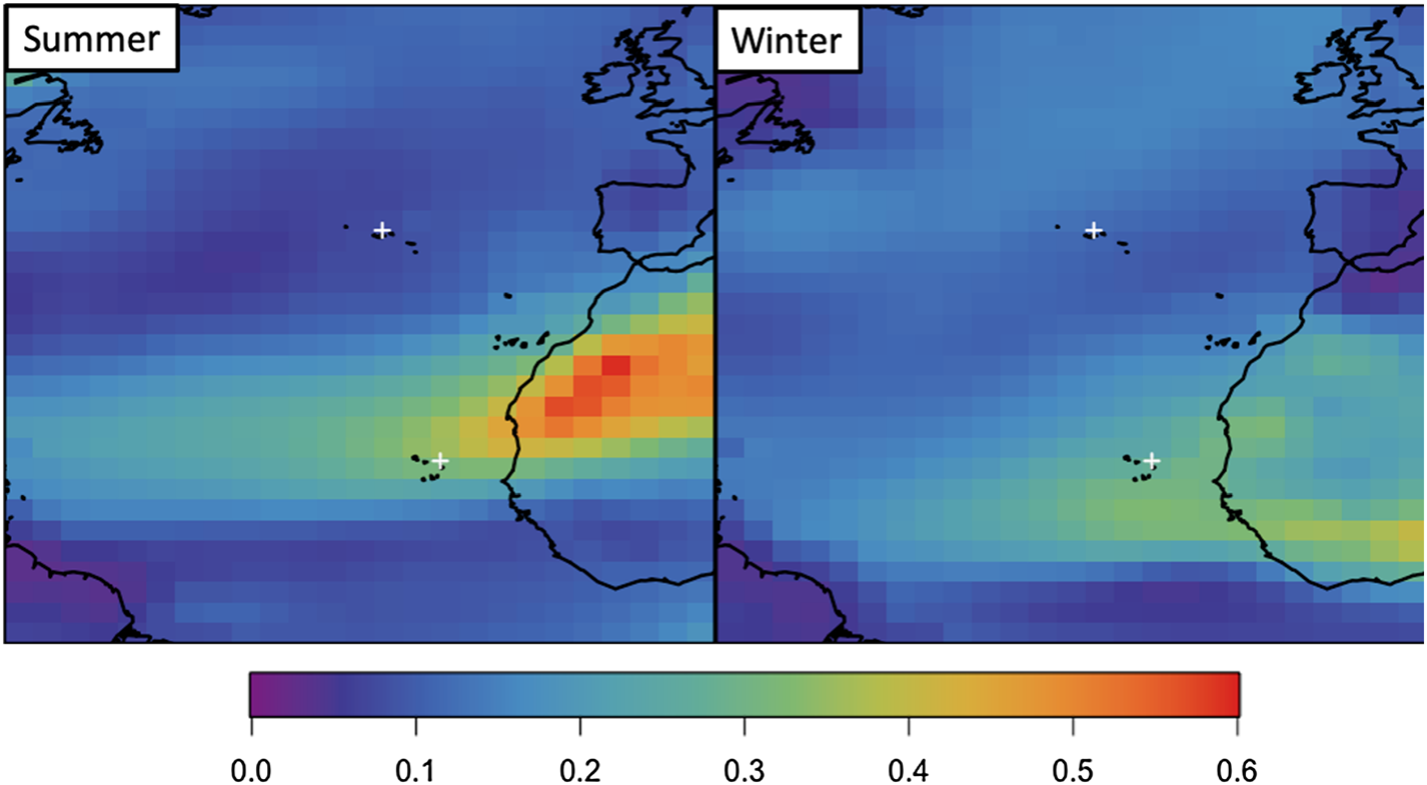
Average seasonal simulated AOD over the North Atlantic for the year 2013 (summer: June, July, and August; winter: December, January, and February). White crosses mark the locations of the island AERONET observatories described in section 2.2.

Fire emissions are from the Global Fire Emissions Database v4 (GFED4, Randerson et al., 2017), and shipping is from the EMEP inventory (Vestreng et al., 2007), both of which are matched to the meteorological year of the simulation. We use aircraft emissions from the AEIC inventory for the year 2005 (Stettler et al., 2011), and global anthropogenic emissions are from the EDGAR v4.2 (EC‐JRC/PBL, European Commission, Joint Research Centre (JRC)/Netherlands Environmental Assessment Agency (PBL)) emissions inventory for the year 2008. Over North America, we use the NEI11 emissions inventory as implemented in Travis et al. (2016) matched to the year of the simulation, and we use the MIX emissions inventory over China for the year 2010 (Li et al., 2014). BC and organic carbon aerosol emissions in this work are from Bond et al. (2007) matched to the meteorological year of the simulation up to the year 2012. The model is driven with GEOS‐5 meteorology for 4 years (2010–2013), with relevant output saved over the North Atlantic domain (0–60°N, 60°W to 0°E; see Figure 1); we focus much of the analysis on the year 2013. This period broadly captures a variety of environmental features and dynamical conditions likely to be important for aerosol transport across the North Atlantic (e.g., volcanic emissions and variability in the North Atlantic Oscillation) and is thus generally representative of the climatology.

### 2.2 AERONET Island Observatories

We use observations from two Aerosol Robotic Network (AERONET; Holben et al., 1998; Smirnov et al., 2009) island sites in this work. AERONET is a network of sun photometers that report AOD every 15 min at multiple wavelengths in the visible and near‐infrared. Here, AERONET observed AOD is interpolated to match the GEOS‐Chem simulated AOD at 550 nm, similar to previous work (e.g., Ridley et al., 2012, 2014). Estimated observational errors are generally quite low, less than 0.02 (Holben et al., 1998). All observations shown in this study are from the Level 2 AOD products, which are calibrated, cloud screened, and manually inspected for data quality prior to being made available (Smirnov et al., 2000). The island sites used here are located on the islands of Cape Verde and Graciosa, shown as the white crosses in Figure 1. Both report mean AOD every 3 hours and are located far from large terrestrial source regions. At each site, less than 1% of all observations reported are less than the observational error (0.4% at Graciosa and 0.07% at Cape Verde).

The Graciosa location is operated by the U.S. Department of Energy Atmospheric Radiation Measurement Eastern North Atlantic (ENA) user facility. Observations of AOD began on the Graciosa island in 2009, persisted for 2 years, and then came online once again at what is now the current ENA user facility in late 2013. The site is located at 15 m above sea level, approximately 1,200 km from mainland Europe. The Cape Verde island site is operated by the Laboratoire d'Optique Atmosphèrique, Université des Sciences et Technologies de Lille. Observations of AOD have been made regularly at this location since 1994. The site is located at 60 m above sea level, approximately 570 km off the coast of Senegal, Africa.

## 3 Results

### 3.1 Simulated Variability in Atlantic AOD

Seasonal simulated AOD across the entire North Atlantic region is shown in Figure 1 for the year 2013. AOD values range from near zero to greater than 0.6 over continental source regions. In general, variability is large across the region, with seasonal changes in magnitude greater than a factor of 2 at any given location. This spatiotemporal variability is largely dependent on seasonal emissions and transport of dust, though there is an additional influence of long‐range transport from North America modulated by seasonal meteorological conditions (e.g., the position of the jet stream). Other simulated years (2010–2012) look quite similar to 2013, with seasonal average values over the ocean varying by ~ ±0.05 and those over the large source region in Northern Africa by up to 0.15.

**Figure 2:**
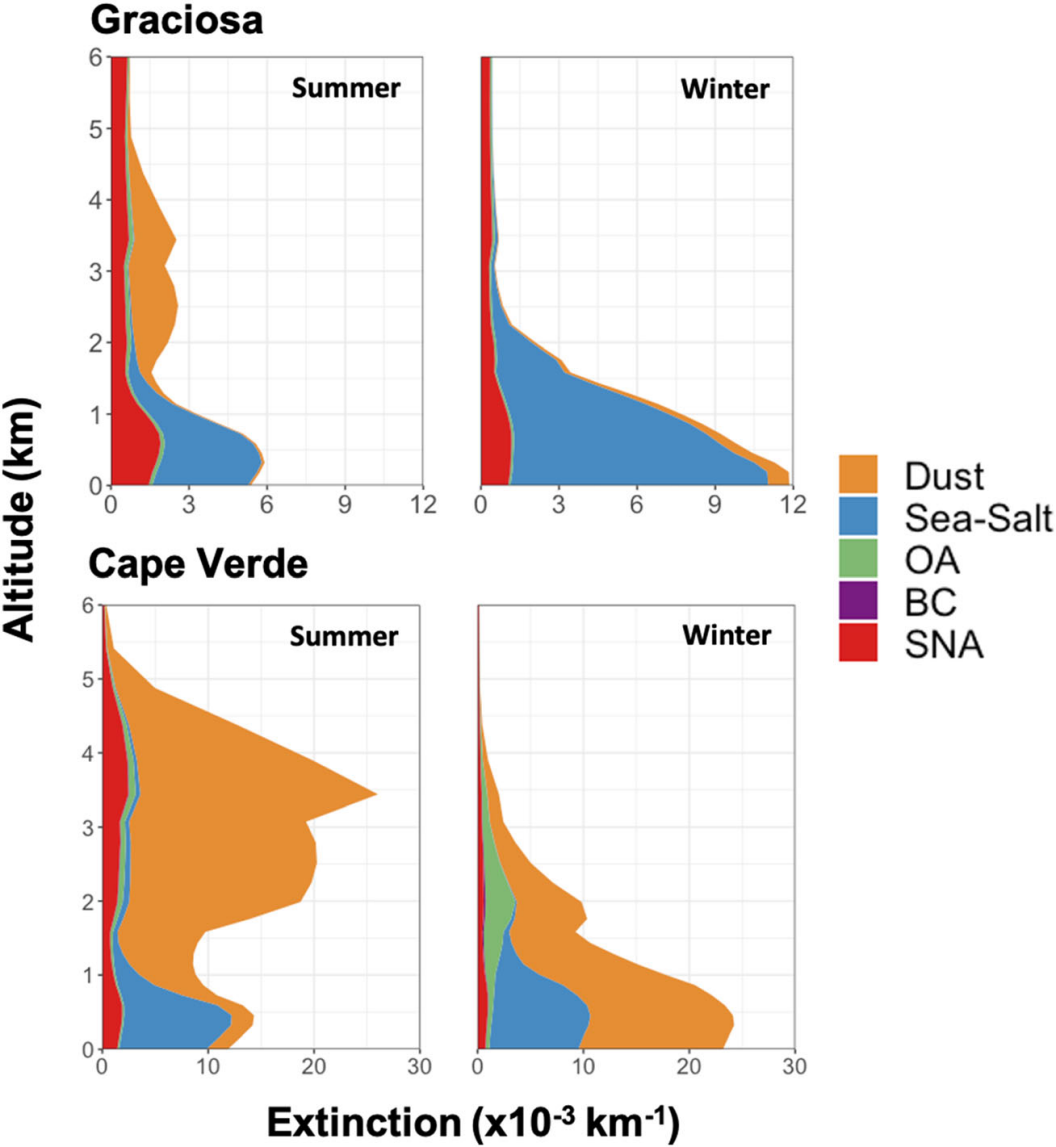
The simulated seasonal average speciated aerosol vertical extinction profile at Graciosa (top) and Cape Verde (bottom) for summer (left) and winter (right) of 2013. The fill color corresponds to the aerosol type.

To better understand the spatiotemporal variability shown in Figure 1, we investigate the simulated speciated aerosol extinction vertically at both island sites, shown in Figure 2 for the year 2013. Over both Cape Verde and the Azores, the largest contributor to aerosol extinction is from natural sources for all seasons, largely sea salt and dust. Extinction at the surface is dominated by sea salt at both locations, with absolute magnitudes varying seasonally with temperature and wind speed. Over Graciosa, the extinction largely follows the local source of sea salt emissions (highest in winter, agreeing with surface observations from Logan et al., 2014). There is additional extinction associated with anthropogenic SNA that is relatively constant throughout the year and related to the long‐range transport of anthropogenic emissions from North America. Over Cape Verde, the variability is dominated by dust aerosol. The vertical distribution changes from dust extinction aloft in the summer months to near‐surface dust extinction in the winter months. This seasonal variability in extinction at Cape Verde relates directly to the two seasonal modes of dust emissions from Africa (e.g., Engelstaedter et al., 2006), where dust emissions from the Sahara peak during the Northern Hemispheric summer months and maximum dust emissions from the Sahel occur during the Northern Hemispheric winter. Across the simulation period (2010–2013), both locations show interannual variability in aerosol extinction of generally less than 30% (i.e., 0.001 km^−1^ for Graciosa and 0.05 km^−1^ for Cape Verde) with the largest interannual variability corresponding to the largest total extinction magnitudes (e.g., near‐surface at the Graciosa site).

**Figure 3:**
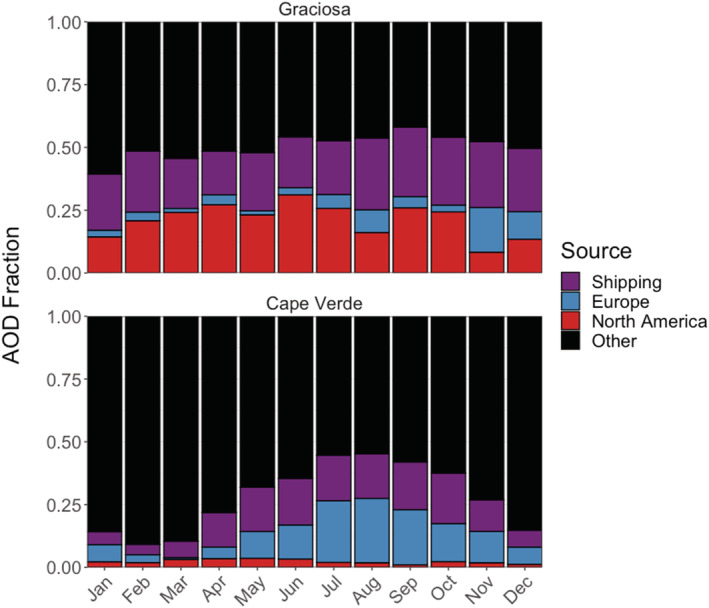
Fractional source contributions to AOD from non‐sea salt and dust aerosol per month in 2013: Graciosa (top) and Cape Verde (bottom). Red: anthropogenic North American sources, blue: anthropogenic European sources, purple: shipping. The black “other” bars indicate sources from the rest of the world, volcanoes, and the oceans.

We perform a set of sensitivity experiments to further explore the AOD variability at both the Graciosa and Cape Verde locations. Given that the majority of the variability at each location comes from local or natural sources (sea salt and dust), we design these sensitivity experiments to investigate sources that are more likely related to long‐range anthropogenic transport. We explore three scenarios where we remove the emissions from shipping, anthropogenic emissions over North America, and anthropogenic emissions over Europe. In this way, we isolate the effect of different non‐sea salt and dust emissions sources on total AOD variability. The contribution of these sources to AOD per month at both island locations is shown in Figure 3. These sources vary seasonally, with the largest seasonal variability over Cape Verde, due primarily to an enhanced influence from shipping and European anthropogenic emissions from June through October. The relative contributions of different sources to AOD over Graciosa is fairly consistent, with a small increase in late year emissions recirculated from Europe. In general, over both locations, more than half of all the AOD fraction comes from sources denoted as “Other” in Figure 3, such as volcanic emissions, non‐sea salt ocean emissions (e.g., marine OA), biomass burning, and anthropogenic emissions from the rest of the world outside North America and Europe. For Cape Verde specifically, anthropogenic and biomass burning emissions from Africa make up a substantial component of the “Other” sources in Figure 3.

### 3.2 Comparison to AERONET Island Observations

**Figure 4:**
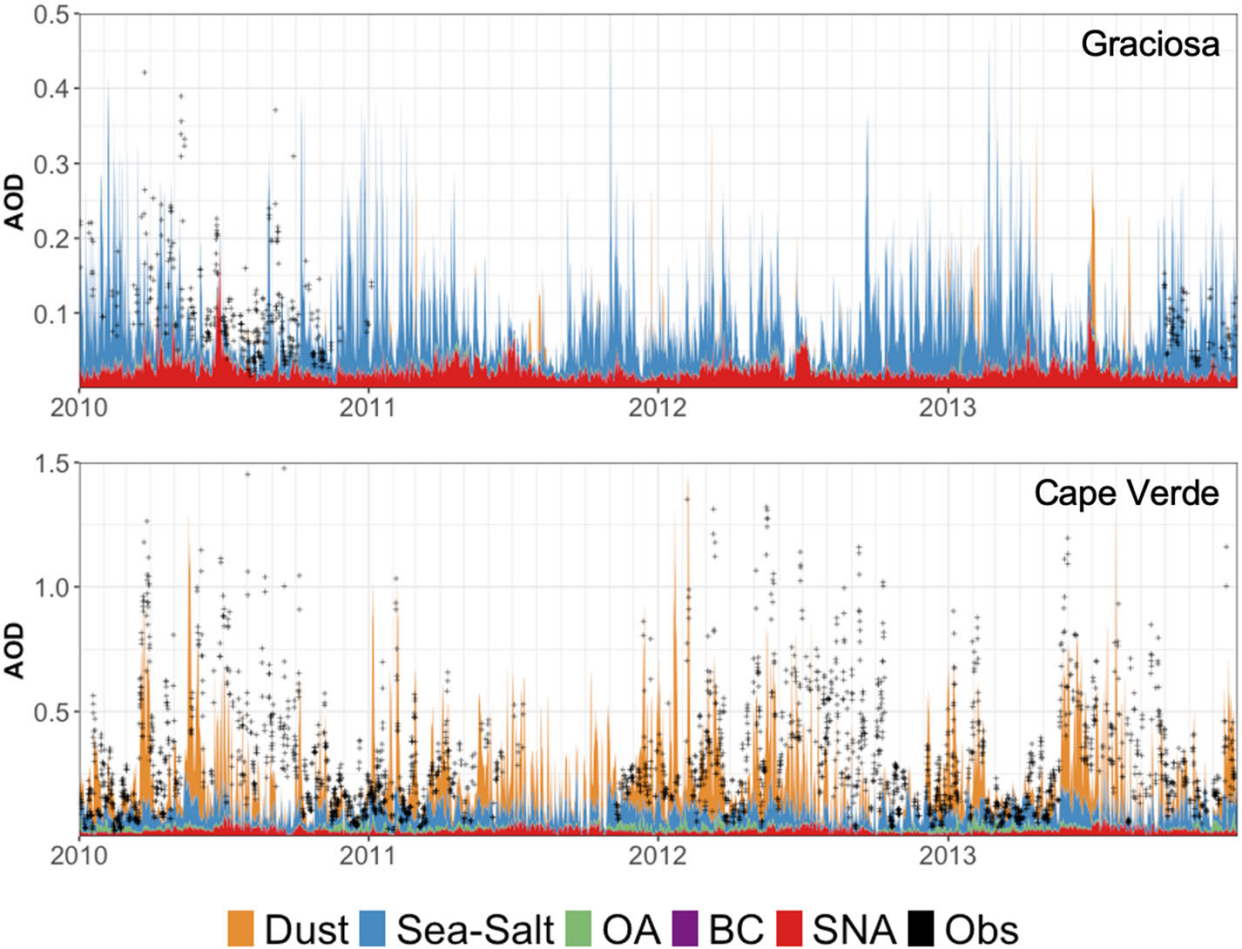
Time series of 3‐hourly AOD at Graciosa (top) and Cape Verde (bottom) from 2010 through the end of 2013. The fill color corresponds to 3‐hourly simulated aerosol type, and the black crosses correspond to observed AOD. Annual tick marks correspond to 1 January.

We assess the GEOS‐Chem model performance using observations from the AERONET sites at both Graciosa and Cape Verde. A time‐series comparison is shown in Figure 4, where both locations demonstrate a consistent background AOD of sea salt aerosol extinction in the model of 0.06 ± 0.05 and 0.06 ± 0.03 for Graciosa and Cape Verde, respectively. The simulated AOD generally agrees with the observations (within ±50%, *R* = 0.56) and captures the dominant year to year variability at both sites, suggesting no major model errors in the representation of the aerosol lifecycle. The model indicates that at Graciosa, the AOD is usually dominated by this local sea salt aerosol, though elevated concentrations of dust and SNA aerosol dominate specific events. The speciation of extinction at Cape Verde is more consistent, dominated by dust aerosol. At both locations, there is large variability on weekly and interannual time scales, which is consistent with plume transport processes.

**Figure 5:**
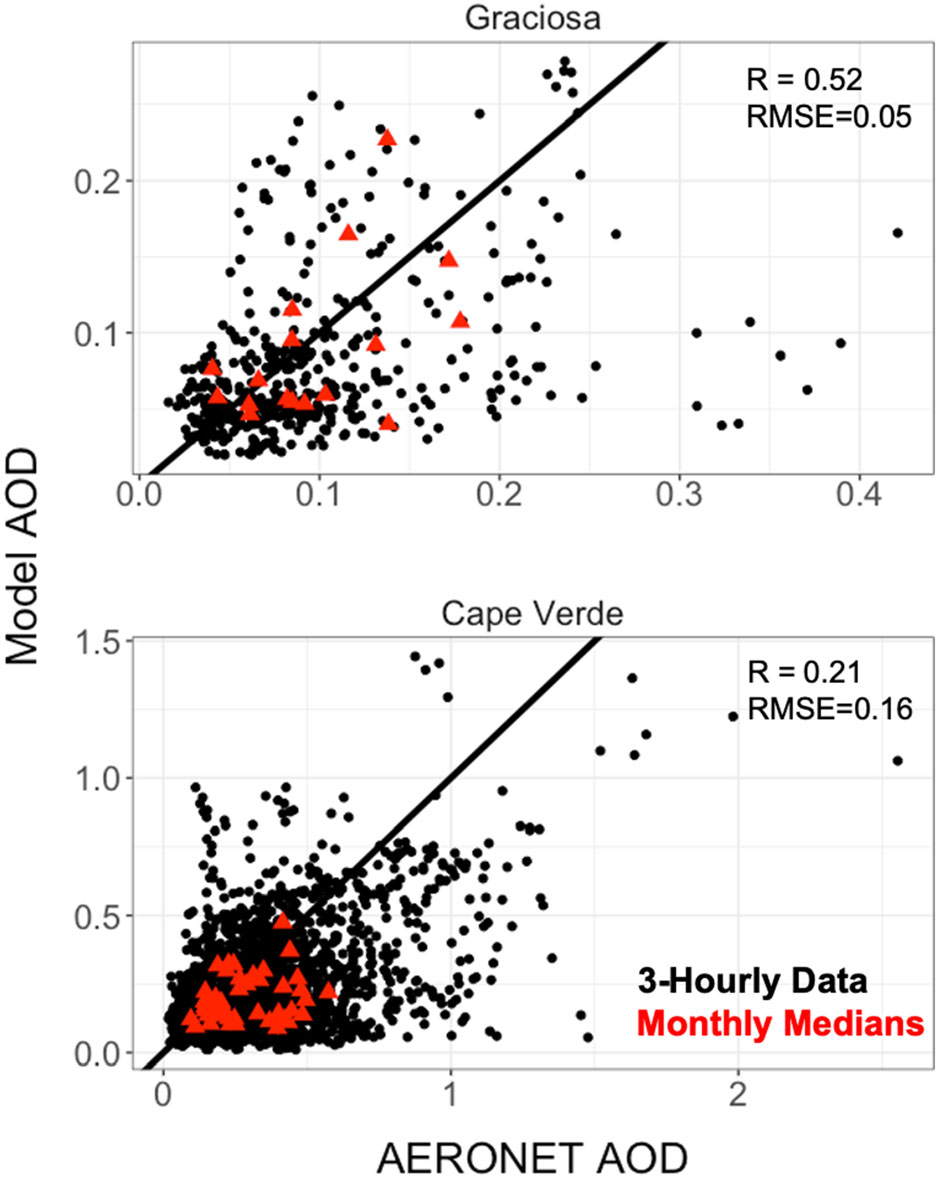
Scatterplot comparison of the GEOS‐Chem model simulated AOD and AERONET observations at 3‐hourly resolution for both Graciosa (top) and Cape Verde (bottom) from 2011–2013. Black points are individual observations, and red triangles are the monthly median values. The 1:1 line is shown in black, and monthly median statistics are shown in each panel.

Related to Figure 4, scatterplots of the model total AOD and AERONET observations at both sites are shown in Figure 5. Individual observations are shown as the black points, where monthly medians are shown in red triangles. Overall, the monthly median comparisons have an R of 0.48 and an RMSE of 0.13. Model performance in the monthly median over the Graciosa site is better (*R* = 0.52, RMSE = 0.05) than over Cape Verde (*R* = 0.21, RMSE = 0.16). As evident in the summary statistics and the fairly wide scatter throughout Figure 5, the fine temporal heterogeneity in observations is not well captured. Given that the simulations calculate gridcell averages at a spatial resolution of 2° × 2.5°, we would not expect the very high observed values to be well represented by the model, as fine‐scale plume structure is smoothed out (e.g., Eastham & Jacob, 2017). This is exemplified by the cluster of large model underestimates when AERONET AOD is greater than 0.3 over Graciosa. These are all points from three different plume fronts that were not captured in GEOS‐Chem (see Figure 4). To better constrain the extent to which this limited model representation impacts the ability to simulate aerosol extinction, there is a need for long‐term observations at both sites. This is particularly the case for the Graciosa site, where the contribution of different aerosol species is more variable and there are fewer reported observations of AOD.

### 3.3 Additional Constraints From a High‐Altitude Pico Observatory

**Figure 6:**
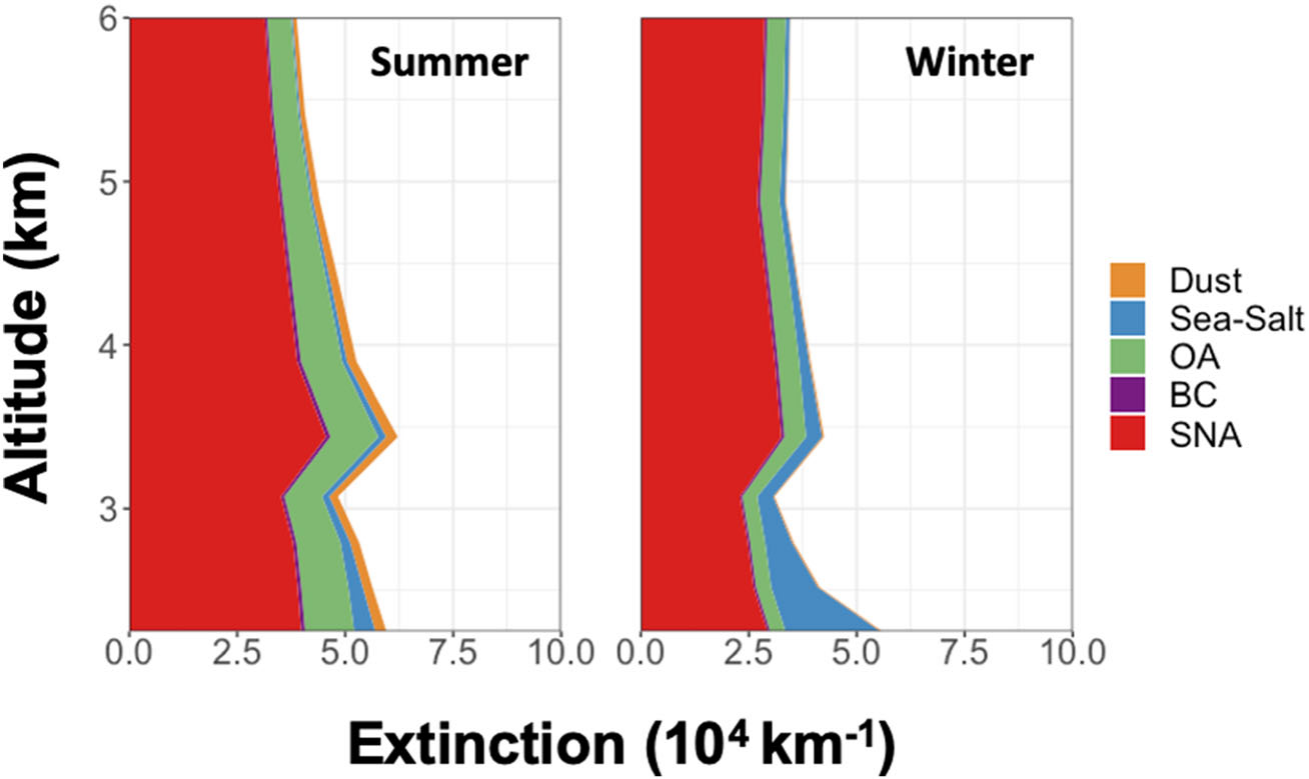
The simulated seasonal average vertical speciated extinction profile above Pico Mountain in the Azores for summer (left) and winter (right). The fill color corresponds to aerosol type. Note that the*y*axis originates at the altitude of observations on Pico Mountain (2.225 km).

In situ observations at the higher elevation site at Pico Mountain in the Azores, near Graciosa, have previously been used to constrain long‐range transport of pollutants (e.g., Helmig et al., 2008; Kumar et al., 2013; Val Martin et al., 2008), though long‐term sun photometer observations do not currently exist at this location. At 2,225 m above sea level, this site is frequently above the boundary layer, thus avoiding the large local sources of sea salt and predominantly samples air masses transported from other regions through the free troposphere. Analogous to Figure 2, in Figure 6, we plot the simulated vertical speciated extinction at Pico Mountain Observatory. In this region, the AOD is substantially lower than the near sea‐level sites, and nearly all aerosol extinction is due to SNA aerosol and (to a lesser extent) OA. There are no strong sources of these aerosol species locally; they are all associated with long‐range transport from landmasses. This reinforces the utility of monitoring at high‐altitude sites, such as Pico Mountain, for identifying long‐range transport of aerosols as well as gas‐phase pollutants.

## 4 Island Observations as Predictors of Imported Aerosol to Europe

In addition to their value as constraints on air transported from far upwind, we explore the value of these island observations as predictors of aerosol further downwind, over continental Europe. Here, we focus on the average background imported aerosol, as opposed to the more specific case of certain meteorological conditions advecting large plumes directly to continental Europe (e.g., Prodi & Fea, 1979). We define imported aerosol as aerosol just upwind of a given location, following the dominant westerly flow in the North Atlantic region. As a proof of concept, we use a gradient boosted tree‐based machine learning regression model known as XGBoost (Chen & Guestrin, 2016) to explore if there is a relationship between AOD at the island sites and AOD just upwind of Lisbon, the largest urban region (metropolitan area population >2.5 million) on the western boundary of continental Europe. Due to the limited availability of sufficient direct observations for developing the XGBoost regression model, we use the GEOS‐Chem model output for analysis in this section, and we posit that, given the general model skill in reproducing observed AOD over the Atlantic, these conclusions may be representative of real‐world conditions.

The XGBoost model is a supervised learning tree‐based model that trains an ensemble of decision trees using a method known as “Gradient Boosting” (Friedman, 2001). We select this specific regression model primarily because it has been shown to be a good prediction method for atmospheric composition (Ivatt & Evans, 2020), yields predictions for reasons that are human interpretable, and it does not require a very large number of training observations of other machine learning models like neural networks (Rasp et al., 2018; Silva et al., 2019). We also find that XGBoost provided the best performance (highest R and lowest Root Mean Squared Error) over other methods for prediction, including Ridge, Elasticnet, and Lasso regression (Hastie et al., 2001), as well as simple ordinary least squares multiple linear regression.

**Figure 7:**
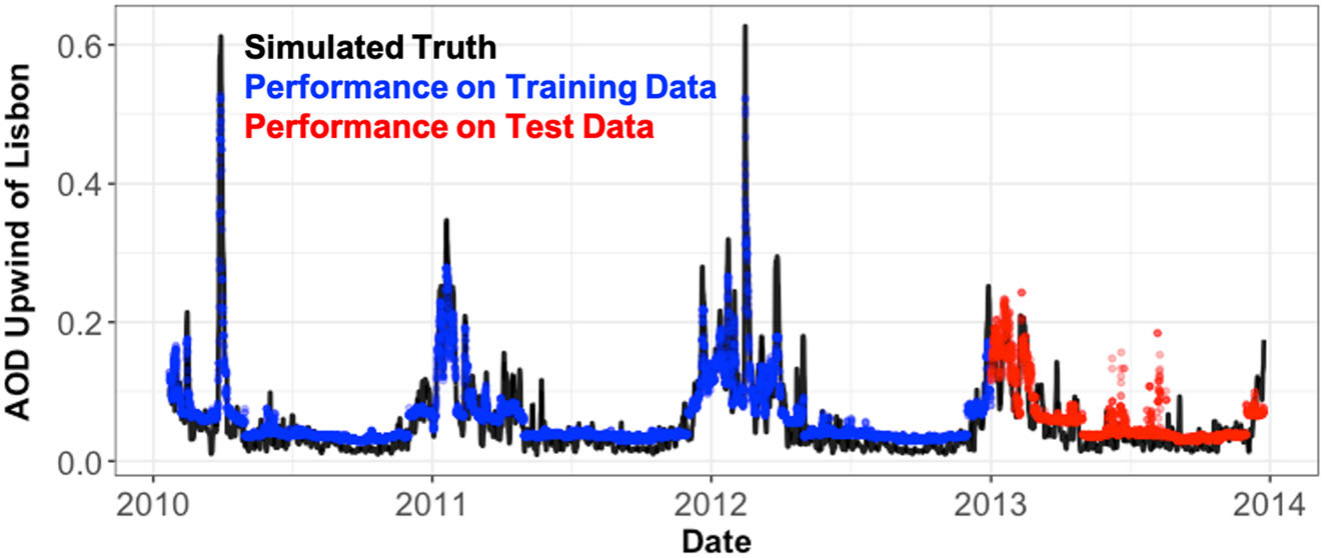
XGBoost reconstruction of simulated AOD upwind of Lisbon using only island observatory information from simulated observations at Graciosa and Cape Verde.

We evaluate if the 24‐hour median imported column AOD to Lisbon can be predicted 1 week in advance using a 14‐day time series of 3‐hourly simulated island observations at the Graciosa and Cape Verde locations. We use these 224 total observations as predictors, along with temporal information of the observations (month of the year), to forecast the median imported AOD 7 days after the final observation was made. In the model space, we define the imported aerosol extinction as the aerosol extinction in the model gridcell just west of the gridcell containing Lisbon. We split the data set into two components by time: a training data set (2010–2012) and a test data set (2013). This enables a robust assessment of the model confidence and uncertainty. The XGBoost model reproduces the full GEOS‐Chem simulation very well for the training data set, consistent with many machine learning approaches. Since good performance on training data is not necessarily related to performance on unknown model inputs, we only describe the performance on a test set (the year 2013) from here forward. In the test data, the XGBoost model captures the broad variability of the evaluation data (*R* = 0.78) and reproduces the overall magnitude of the full model well (RMSE = 0.03). This correlation is much higher than the correlation of the GEOS‐Chem simulated observations with the imported AOD alone (Cape Verde *R* = 0.36 and Graciosa *R* = 0.3) and indicates a potential value in these island observations as non‐linear constraints on European air quality. Much of the missing variability in Figure 7 is likely due to additional local processes and regional meteorology, which cannot be captured from observations so far upwind.

To better understand the potential value is being added by the island observations, we investigate the so‐called “gain” values associated with each observed model input, or “feature” (i.e., location and temporal lag), used in the boosted regression. In this context, gain is a metric for the improvement in accuracy achieved by the addition of a given feature (e.g., AOD observations at a given site and temporal lag) where it is used in the model. The most important features are related to the temporal context (month) of the simulated observations, likely capturing the large‐scale seasonality in the imported AOD (higher in local winter). Following the monthly gain values, Cape Verde is the most common location of the top 30 ranked gain features in the prediction, followed by Graciosa island. In general, the most important AOD lag times were larger over Cape Verde (usually >7 days) than over the site in the Azores (usually <7 days), consistent with shorter transport times from the Azores to Lisbon, compared to the transit from Cape Verde to the same destination. This importance ranking selecting both the month feature and lagged simulated observations at Cape Verde is consistent with large imported aerosol events being dominated by seasonal dust transport from Africa in the European winter months. However, the importance of the other island location points to complementary information provided by this site for forecasting imported aerosol to European cities downwind.

We investigate the potential added value of long‐term AOD observations from an AERONET observatory on Pico Mountain by rerunning the XGBoost predictor using lagged AOD at the location of Pico Mountain (see section 3.3). We find that the additional information from this hypothetical long‐term AERONET observatory very marginally impacts the imported AOD predictive skill (e.g., 2% relative reduction in RMSE and correlation). However, the information from Pico Mountain is deemed to be important as interpreted through the presence of Pico Mountain features in the top 30 ranked gain features.

We perform an additional prediction task to explore how well AOD can be used to predict downwind simulated surface aerosol concentrations, rather than AOD. The prediction technique and setup are identical to that described for AOD, except model surface concentrations of total aerosol mass are predicted (instead of AOD) as a function of AOD at the island sites. The XGBoost model performs similarly well to the AOD prediction (*R* = 0.7, RMSE = 7.8 μg m^−3^), demonstrating the potential for these island sites to provide relevant information for forecasting surface, and thus human health exposure‐relevant, imported aerosol concentrations.

It is important to note that aerosol abundances (and thus AOD) are highly influenced by meteorological conditions, and without explicitly including this information (e.g., through data assimilation), it is difficult to fully assess the utility of these data for forecasting imported aerosol to Europe. This model is potentially overconfident due to the relatively consistent seasonal pattern apparent in the training data. If a year with a different relative seasonal pattern were weighted heavily in model training, or if a different set of input parameters were selected (e.g., a different temporal lag), the performance of the model may be worse on the test set. Additionally, the machine learning model takes as input average AOD across a 2° × 2.5° grid box, which does not perfectly represent the total variability seen in the point observations (see section 3.2). Higher‐resolution GEOS‐Chem simulations or direct observations of AOD at all three sites and the inflow domain would be a more direct assessment of the influence of how these higher resolution processes impact the machine learning model performance. Despite these caveats, the results shown in Figure 7 do demonstrate that the observations at these island sites in the North Atlantic contain information relevant for forecasting imported aerosol to European cities.

## 5 Conclusions

The work presented in this manuscript explores the constraints that island observatories can provide on the long‐range transport of aerosols over the oceans, a region with a dearth of in situ observations. We focus on AOD, a measurement that is largely automated at a global network of sun photometers, and thus provides long‐term atmospheric monitoring. Over the North Atlantic, simulated AOD is dominated by local emissions of sea salt and intercontinental transport of dust, with short‐term variability controlled primarily by meteorology. Given this variability and dominant local sources, there is strong value in the complementarity of the two different island sites at Graciosa and Cape Verde for exploring the influence of different sources on AOD throughout the region, constraining the aerosol lifecycle, and evaluating chemical transport model skill. We show that though these sites are located far from the continents, they provide valuable information for predicting imported aerosol extinction above the European continent. Though we focus here on using automated remote sensing observations of AOD to get insight into key transport processes, long‐term continuous in situ measurements of aerosol composition would allow for improved constraints on the variability and trends of aerosol sources across the North Atlantic. Additional work could explore model fidelity by using speciated aerosol mass concentrations from short‐term aircraft campaigns. Integrating these point measurements with machine learning methods could potentially be used to improve air quality forecasting in regions across the globe.

Long‐term measurements like those available from the AERONET island sites are critical to understanding trends, causes, and ultimate impacts of AOD worldwide. Measurements at a few locations can provide critical insight into regional and hemispheric processes and ultimate impacts on air quality and climate. In a changing Earth system that is in part driven by changes in aerosol abundance, it is ever critical to understand these processes.

## Conflict of Interest

The authors declare no conflict of interest.

## Data Availability

The AERONET AOD observations are available at https://aeronet.gsfc.nasa.gov/cgi‐bin/data_display_aod_v3?site=ARM_Graciosa&nachal=2&level=1&place_code=10 and https://aeronet.gsfc.nasa.gov/cgi‐bin/data_display_aod_v3?site=Capo_Verde&nachal=2&level=1&place_code=10 for Graciosa and Cape Verde, respectively. The GEOS‐Chem v11–01 code used in this work is available at https://doi.org/10.5281/zenodo.3995574.
